# Telemedicine-based exercise intervention in cancer survivors: a non-randomized controlled trial

**DOI:** 10.1038/s41598-024-83846-x

**Published:** 2024-12-23

**Authors:** Verena Krell, Johanna Porst, Lorena Hafermann, Jessica Kuhn, Franziska Greiß, Claudia Römer, Bernd Wolfarth

**Affiliations:** 1 Department of Sports Medicine, Charité – Universitätsmedizin Berlin, Berlin, Germany; 2 Department of Sports Medicine, Institute of Sports Science, Humboldt-Universität zu Berlin, Berlin, Germany; 3 Institute of Biometry and Clinical Epidemiology, Charité – Universitätsmedizin Berlin, Corporate Member of Freie Universität and Humboldt-Universität zu Berlin, Berlin, Germany; 4 Department of Pediatrics, Division of Oncology and Hematology, Charité – Universitätsmedizin Berlin, Berlin, Germany

**Keywords:** Exercise oncology, Cancer survivors, Wearable activity tracker, COVID-19, Controlled study, Supportive cancer care, Cancer, Quality of life, Lifestyle modification, Oncology

## Abstract

Cancer survivors (CS) often experience treatment-related side effects, such as fatigue, and have reduced physical function. Regular physical activity has been demonstrated to reduce these symptoms and improve cardiopulmonary fitness. Digital solutions are needed to optimize exercise options for CS in aftercare, especially given the significant limitations during the Covid-19 pandemic. This two-armed, non-randomized, controlled intervention study for CS aims to investigate whether a telemedicine-based exercise intervention is as effective as the current standard of care for oncological exercise therapy in aftercare. Patients in the intervention group (*n* = 61) performed a telemedicine-based exercise program (TE) and patients in the control group (*n* = 31) participated in an existing rehabilitation sports group (RG) over a six-month intervention period. The primary outcome was cardiopulmonary fitness measured by VO_2_peak; secondary outcomes included quality of life (QoL), fatigue, and physical activity. A non-inferiority analysis was performed with a predefined non-inferiority margin for relative VO_2_peak of -1.50 ml/min/kg. Although TE demonstrated a slight advantage in relative VO_2_peak compared to RG (adjusted mean difference of 0.55 ml/min/kg [95% CI: -2.74; 3.84]), the non-inferiority was not statistically significant. Nevertheless, the implementation of a telemedicine-based exercise intervention indicates that individual patients respond well to this type of exercise program and benefit from the intervention, particularly in terms of QoL. Finding an individualized program for each cancer survivor is the overarching goal. A telemedicine-based exercise intervention may be a promising option, particularly for younger patients.

**Supplementary Information:**

The online version contains supplementary material available at 10.1038/s41598-024-83846-x.

## Introduction

The relationship between physical activity (PA) and cancer has been the subject of extensive research. There is compelling evidence for the positive psychological and physiological effects of exercise and PA for individuals dealing with cancer. Numerous randomized controlled trials have shown that regular exercise can reduce cancer- and treatment-related side effects such as fatigue^1–3^, chemotherapy-induced polyneuropathy^4–7^, or lymphedema^8–11^. Overall, PA during treatment improves patients’ quality of life (QoL)^12,13^. Physical activity and regular exercise are also associated with a better prognosis. A dose-response meta-analysis of large prospective studies by Garcia et al. showed that higher activity levels were associated with a lower risk of cancer^14^. Potential mechanisms linking exercise and cancer have been widely discussed^15^but are not conclusively known. Based on this evidence, recommendations for PA have been developed. In addition to the general World Health Organization (WHO) recommendations for PA^16^, the American College of Sports Medicine (ACSM) Roundtable has developed guidelines specifically for cancer survivors (CS)^17^. These WHO and ACSM guidelines agree in recommending at least 150 min of moderate-to-vigorous aerobic physical activity plus two to three sessions of resistance training per week. However, many cancer patients do not meet these recommendations^18,19^. Another issue is that many side effects of cancer treatment can develop into long-term consequences. The results of Woopen et al. show that 24% of long-term ovarian cancer survivors (survival > 5 years) continue to experience cancer-related fatigue years after diagnosis^20^. Therefore, high-quality exercise therapy adapted to the specific needs of CS is of particular importance in aftercare.

In Germany, the main form of exercise therapy offered for CS are so-called rehabilitation sports groups^21^. Rehabilitation sport is a prescribed exercise activity as a supplement to medical rehabilitation under the guidance of a specialized exercise instructor. It is intended to promote lifelong physical activity and thus support long-term social and occupational reintegration. The statutory providers of rehabilitation sports groups are sports clubs. The costs are covered by the statutory health insurance^21^. In practice, there are several challenges in Germany: First of all, existing rehabilitation sports groups are often not specifically designed for cancer patients, but encompass a range of other medical conditions such as coronary heart disease, diabetes, or respiratory diseases. A survey by Müller et al. shows, that 88% of courses in Berlin, Germany, are aimed at people with orthopedic problems and only 1% at CS^22^. Tailored therapy for the specific needs of CS is therefore rare. The implementation of these courses shows that comprehensive coverage cannot be guaranteed: In densely populated cities such as Berlin, there are not enough groups for the large number of people seeking treatment, as people with different conditions compete for the same courses. As a result, many patients face long waits and end up in mixed groups, where their specific needs may not be adequately addressed. In rural areas, the challenges are long travel distances and the lack of comprehensive coverage, and as a result, many CS have no access to a rehabilitation sports group at all.

These challenges raise the question of how to use technology and digital media in cancer exercise therapy. Previous research concludes that broad-reach, non-face-to-face interventions are a potential way to increase access to cancer rehabilitation^23,24^. But, a review by Groen et al. indicated that distance-based physical activity behavior change interventions, which utilized only print and telephone methods, have had only negligible to small effects among CS^25^. The most studied types of interventions have been digital behavior change programs and lifestyle change interventions^26^. Among exercise interventions, web-based exercise interventions are the most common^27^. A randomized controlled trial by Galiano-Castillo et al. showed improvements in QoL, pain, muscle strength, and fatigue with an internet-based exercise intervention^28^. Wearable activity trackers (AT) are another technological option that is becoming increasingly important alongside web-based behavior change programs. Previous work has demonstrated the utility and acceptability of AT in older adults^29,30^and CS^31,32^. To date, most work has focused on improving PA by use of AT^33–36^.

The aim of this study is to investigate whether a telemedicine-based exercise intervention is as effective as the current standard of care for oncological exercise therapy in aftercare. In contrast to previous studies, the AT will not only be worn by the CS and evaluated at the end of the study, but will also serve as an accompanying feedback mechanism for both the patient and the instructor. In particular, the effect of AT as an integral part of the exercise intervention on cardiopulmonary fitness as measured by relative VO_2_peak will be investigated. With the increasing availability of digital devices and services, there is a need to explore ways of using AT in the context of aftercare for cancer patients. The overall goal is to consistently improve the range of exercise therapy options for CS.

## Methods

### Study design

This is a two-armed, non-randomized, controlled intervention trial for CS. After baseline (V0), participants either chose a telemedicine-based exercise intervention (TE) or participated in an existing rehabilitation sports group (RG). The intervention period was six months. After the interim evaluation (V1), no additional exercise instructions were given during a six-month follow-up. The study was coordinated at the Department of Sports Medicine, Charité - Universitätsmedizin Berlin (Berlin, Germany), it was performed in compliance with the Declaration of Helsinki and the study protocol was approved by the Ethics Committee of Humboldt-Universität zu Berlin (HU-KSBF-EK_2018_0006).

### Participants and recruitment

The study included CS in curative care whose acute therapy (e.g., surgery, chemotherapy, and/or radiotherapy) had been completed for at least six weeks. Other inclusion criteria were a minimum load capacity of 50 watts on a bicycle ergometer and medical clearance for participation. As the study was funded by the health insurance company AOK Nordost, participants had to be insured accordingly. Eligible participants had to confirm their consent to participate in the study. It was recruited through various departments of the Charité - Universitätsmedizin Berlin (e.g., Department of Gynecology with Breast Center), other clinics, and resident oncologists (mainly in Berlin, Germany), as well as through flyers, websites, and press releases. The recruitment phase lasted from April 2018 to December 2021.

### Exercise interventions

Patients who met the inclusion criteria and provided informed consent were eligible to begin the intervention. The overall goal for all study participants was to meet the WHO recommendations for physical activity and sedentary behaviour^16^. Accordingly, the CS should perform at least 150 min of moderate-intensity physical activity or 75 min of vigorous-intensity physical activity in combination with resistance training twice a week.

Patients of TE received an individualized training plan based on the FITT principle - frequency, intensity, time and type. All parameters were adjusted to the fitness level at the time of enrollment with the goal of *at least* meeting WHO recommendations over the course of the six-month intervention. Therefore, the number of training sessions per week as well as the total duration of the training could vary between participants, depending on their current fitness level. The training consisted of a combination of endurance and strength training. The type of aerobic exercise was adapted to the patients’ interests and previous knowledge and included jogging, (Nordic) walking, (ergometer) cycling or swimming. Based on spiroergometry and a lactate diagnostic test performed at V0, patients were given individual heart rate ranges for their aerobic training. The goal was to provide moderate-intensity endurance training using the extensive duration method in the aerobic range below the individual anaerobic threshold (IAT). In addition, they performed a home-based resistance training twice a week. To start, they first received a prescription for six sessions of physiotherapy to learn exercises using only their body weight. The physiotherapist’s only requirement was that the training be holistic, with a focus on hypertrophy. The exact exercises, number of sets and repetitions were then put together individually. All TE patients received an activity tracker (Fitbit Charge 2) to record steps, active minutes and exercise sessions, including heart rate ranges. These parameters formed the basis for telephone support, which took place approximately once a month. In this way, the frequency, intensity, time and type of individual training could be adjusted according to changing needs and progress of the individual.

Patients of RG attended in different existing rehabilitation sports groups. A group lesson usually lasts between 45 and 60 min. In order to reach the targeted 150 min per week, participants took part in the group twice a week. The study coordinators supported the CS in finding a suitable group close to home and connecting the CS with the instructor of the group. With this step, the patients were discharged to standard care, and study coordinators had no influence on the content of the group training.

### Outcomes and measurements

All outcome measures were assessed at baseline (V0), after six months of intervention (V1), and after six months of follow-up (V2). A self-reported questionnaire collected data on tumor and treatment characteristics as well as data on education and lifestyle. At all three time points, body weight, height, body mass index (BMI), waist-hip ratio (WHR), and body composition using bioelectrical impedance analysis (BIA; InBody 770) was also measured.

Primary outcome measures.

Cardiopulmonary fitness was assessed during a maximal exercise test on an electronically braked cycle ergometer (Ergoselect 100 K, Ergoline). Participants started with an initial load of 25 watts, which was increased by 25 watts every three minutes. The test was terminated by voluntary exhaustion reported by the participant or by the physician for medical reasons. A 12-channel electrocardiogram (ECG; Custo cardio 400, Custo med) was continuously monitored during the test. Blood pressure, lactate concentration and the rate of perceived exertion using the Borg scale (6–20)^37^ were measured respectively at the end of each step. Oxygen uptake was measured with the Cosmed K5 spiroergometry system. Expired gases were collected and analyzed breath by breath to determine VO_2_peak, both absolute and relative to body weight. VO_2_peak was defined as the highest value of oxygen consumption averaged over a 30-second interval within the last two minutes of exercise. Measurements with an implausible drop in VO_2_ of more than 15% during exercise were excluded from analysis. In addition to VO_2_peak, peak power output in watts (absolute and relative to body weight) was recorded.

Secondary outcome measures.

Quality of life (QoL) was measured using the European Organization for Research and Treatment of Cancer Quality of Life Questionnaire-C30 (EORTC QLQ-C30). The QLQ-C30 includes one global health status / QoL scale consisting of one single item, five functional scales consisting of multiple items, and a total of nine symptom scales consisting of both single and multiple items. All scales range in scores from 0 to 100, with higher scores indicating a higher level of response. Thus, a high score on the global QoL or functional scales represents a high level of QoL or functioning, whereas a high score on the symptom scales represents a high level of symptomatology^38^.

Fatigue was assessed using the Functional Assessment of Cancer Therapy-Fatigue (FACT-F). The questionnaire consists of 13 items with a four-point scale. The total score ranges from 0 to 52, with higher scores indicating more severe fatigue^39^. In addition, we used the proposed ICD-10 criteria for cancer-related fatigue, consisting of eleven items, which were developed by Cella et al.^40^ to assess the overall occurrence of fatigue in the study sample. These criteria were met if six or more of the symptoms were present every day or almost every day during a 2-week period in the past month.

Physical activity (PA) was estimated using the validated International Physical Activity Questionnaire – Long Form (IPAQ-L)^41^. The questionnaire asks about activities during the past seven days in five different domains: work, transport, household, yard/garden and leisure activities. For each domain, the number of days and the active minutes per day spent at different intensities (moderate, vigorous) are recorded. Additionally, sedentary activities are reported. The data collected are used to estimate total weekly physical activity. Total PA is reported as metabolic equivalent of task (MET) minutes per week (min/week). According to the IPAQ-L manual^42^, participants can be categorized into one of the following three physical activity levels (PAL): low PAL (< 600 MET min/week), moderate PAL (600–3000 MET min/week) or high PAL (> 3000 MET min/week).

In addition to the IPAQ-L, we used the Fitbit Charge 2 activity tracker (AT) as an objective measure of PA. The AT tracks steps, total active minutes, and minutes of light, moderate, and vigorous activity. It also measures resting heart rate per day and calories burned (total and active burned calories). For data analysis, we followed the recommendation of Migueles et al.^43^ and defined a valid day as at least 10 h of wearing time per day and a valid week as at least 4 valid days. The number of steps is reported as the mean per (valid) day, and the number of active minutes is reported as the mean of the sum per week.

WHO recommendations for PA were verified using both IPAQ-L information and AT data. Minutes of vigorous intensity were counted twice and added to minutes of moderate intensity. A total of at least 150 min was considered to meet the recommendations.

### Statistical analysis

First, all baseline characteristics are presented descriptively stratified by TE and RG group by reporting mean and standard deviation (SD) for continuous variables and absolute and relative frequencies for categorical variables. For skewed variables, the median and the range are also provided.

The primary endpoint was the mean change in relative VO_2_peak between V0 and V1, analyzed using a 95% confidence interval approach based on a linear regression model. Only values identified as valid as described above were used. Non-inferiority of TE to RG could be claimed if the lower limit of the confidence interval for the difference in mean change in VO_2_peak was greater than the predefined margin of -1.50 ml/min/kg. The margin of non-inferiority of -1.50 ml/min/kg was determined *prior to* the analysis and is based on extensive discussion among experts in the field in terms of clinical relevance. The treatment effect was adjusted for age, VO_2_peak, FACT-F, and MET min/week at baseline, which was selected on the basis of a directed acyclic graph (DAG) (see Supplementary Appendix I). All secondary endpoints were described descriptively by means and standard deviations. In addition, non-inferiority tests were performed based on adjusted linear regression models that provide confidence intervals. As there was no adjustment for multiple testing, these can only be interpreted descriptively. The following predefined non-inferiority margins were selected for the secondary endpoints: -5 points for change in QoL, 3 points for change in FACT-F and − 720 MET min/week for change in PA. Similar to the primary endpoint, the adjustment variable was determined on the basis of a DAG; further details are provided in the Supplementary Appendix I.

Missing values for the primary endpoint were imputed using multiple imputation, as there were 13.9% missing data for the primary model. After visualization and discussion it was concluded that the “missing at random” assumption was valid. For multiple imputation, predictive mean matching was used with 10 imputations, and variables related to intervention, diagnosis, cardiopulmonary fitness, fatigue, physical activity, QoL, and demographic information were used to impute the missing values; the list is provided in the Supplementary Appendix II. The software *R*(version: 4.3.1)^44^ was used for the analysis, the *R* package *ggplot2*(version: 3.4.4)^45^ for figures and the *R* package *mice*(version: 3.16)^46^ was used for the multiple imputation.

## Results

**Fig. 1:**
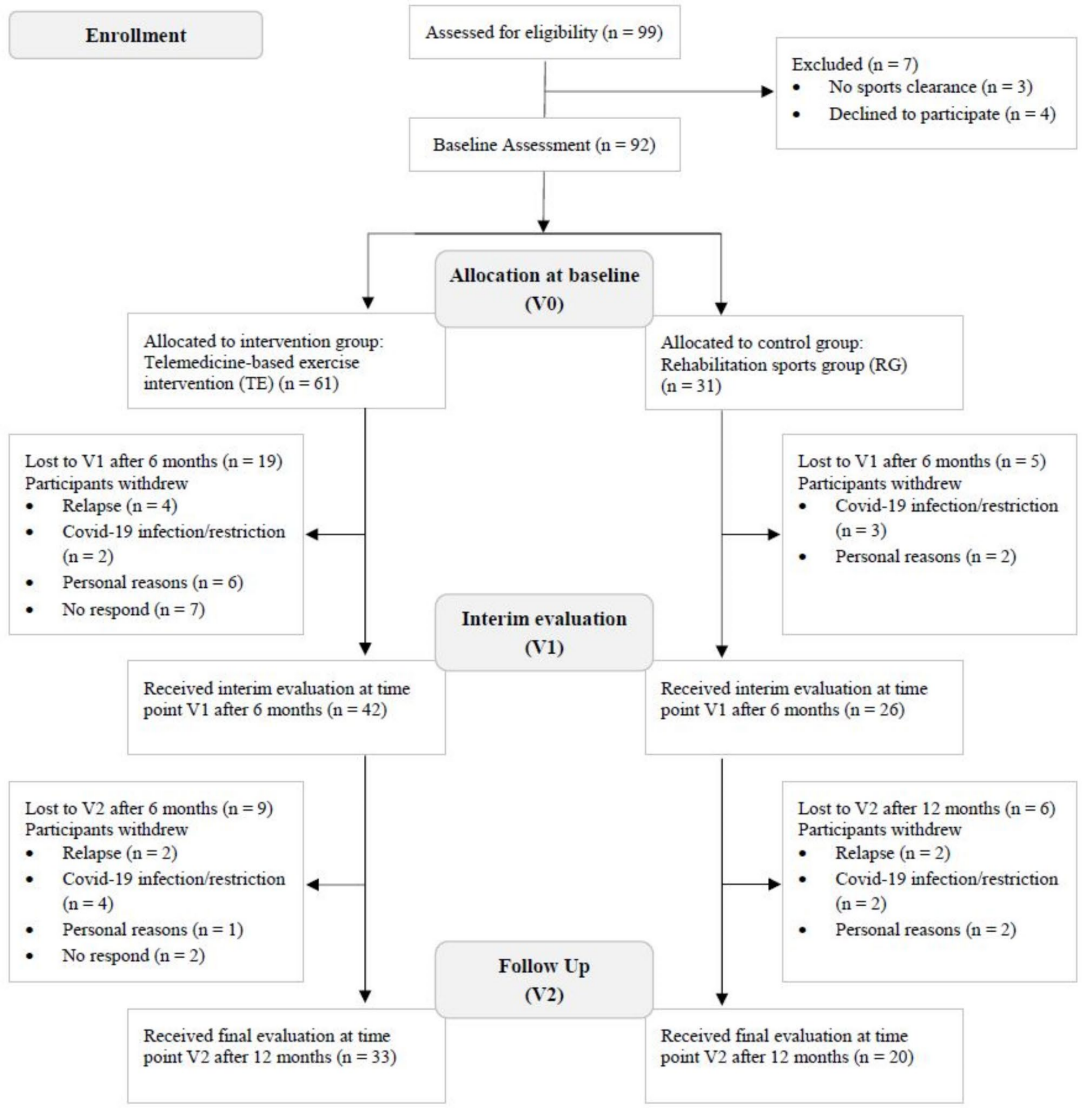
Flowchart from enrollment to analysis including dropouts.

From July 2018 to April 2022, a total of 99 cancer survivors were screened, of which 92 met the inclusion criteria and started the intervention. Sixty-eight patients completed V1 and 53 patients completed V2. All patients who completed V1 were included in the analysis. The overall dropout rate was 42% (*n* = 39) and was higher in TE (46%; *n* = 28) than in RG (35%; *n* = 11). The most common reasons for dropout were Covid-19 infection or pandemic-related restrictions (*n* = 11) and personal reasons (*n* = 11). Figure 1 shows the flowchart from enrollment to analysis, including the number and reasons for dropout.

### Patient characteristics

Due to the patient preference allocation, an uneven distribution was observed (see Table 1), i.e., two-thirds of the participants (*n* = 61; 66%) chose the telemedicine-based exercise intervention (TE). 60% of all participants (*n* = 55) were men, with a higher proportion in RG (*n* = 21; 68%) compared to TE (*n* = 34; 56%). The two groups also differ in terms of age. The overall mean was 53.3 (± 16.5) years, with TE participants being younger (46.9 ± 15.7 years) than RG participants (62.8 ± 12.7 years). There were no differences in body composition between the two groups. The most frequent diagnosis in TE was breast cancer (*n* = 16; 26%), followed by prostate cancer (*n* = 11; 18%) and lymphoma (*n* = 6; 10%). In RG, most frequent diagnosis was prostate cancer (*n* = 13; 42%), followed by breast cancer (*n* = 5; 16%) and lymphoma (*n* = 3; 10%). Consequently, radiotherapy was less common in RG than in TE. 90% of RG (*n* = 27) were unemployed but 65% of TE were full (*n* = 25) or part time (*n* = 11) employed. The mean time since diagnosis was 3.7 (± 3.4) years in both groups with a median of 29 months in TE and 26 months in RG. The range is shifted to the right in RG (range 9–203 months) compared to TE (range 2–162 months). In terms of physical activity level (PAL) at baseline, TE patients were more active than RG patients. According to the IPAQ analysis 83.7% (*n* = 41) of the TE met the WHO recommendation of at least 150 min of moderate physical activity or 75 min of vigorous physical activity per week. In RG, the proportion was 52.9% (*n* = 9) at baseline.

**Table 1:** Patient characteristics at baseline.

	Total (*n* = 92)		TE (*n* = 61)		RG (*n* = 31)	
	n (%)	Mean ± SD	n (%)	Mean ± SD	n (%)	Mean ± SD
**Age (years)** (*n* = 92) Mean ± SD Median (range)	92 92	52.3 ± 16.5 55 (18; 80)	61 61	46.9 ± 15.7 47 (18; 80)	31 31	62.8 ± 12.7 65 (22; 79)
Gender (*n* = 92) female male	37 (40.2) 55 (59.8)		27 (44.3) 34 (55.7)		10 (32.3) 21 (67.7)	
Body composition (*n* = 92) Height (cm) Weight (kg) BMI (kg/m^2^) WHR (waist/hip)	92 92 92 92	171.2 ± 9.3 83.2 ± 20.4 28.2 ± 5.9 0.92 ± 0.10	61 61 61 61	171.4 ± 9.7 83.8 ± 22.0 28.3 ± 6.4 0.90 ± 0.10	31 31 31 31	170.8 ± 8.6 82.2 ± 17.2 28.0 ± 4.8 0.96 ± 0.09
Bioelectrical impedance analysis (*n* = 89) Body fat (%) Muscle mass (%)	89 89	33.5 ± 9.7 36.7 ± 5.6	59 59	32.2 ± 10.3 37.7 ± 5.9	30 30	36.1 ± 7.9 34.9 ± 4.6
Diagnosis (*n* = 92) Prostate-Ca Mamma-Ca Lymphoma Leukemia Colorectal-Ca Testicular-Ca Bladder-Ca Thyroid-Ca Other	24 (26.1) 21 (22.8) 9 (9.8) 5 (5.4) 7 (7.6) 5 (5.4) 5 (5.4) 5 (5.4) 11 (12.0)		11 (18.0) 16 (26.2) 6 (9.8) 3 (4.9) 5 (8.2) 4 (6.6) 4 (6.6) 5 (8.2) 7 (11.5)		13 (41.9) 5 (16.1) 3 (9.7) 2 (6.5) 2 (6.5) 1 (3.2) 1 (3.2) 0 (0) 4 (12.9)	
Months since diagnosis (*n* = 92) Mean ± SD Median (range)	92 92	44.2 ± 40.2 29 (2; 203)	61 61	44.7 ± 38.8 29 (2; 162)	31 31	43.3 ± 43.4 26 (9; 203)
Therapy (*n* = 92) Surgery Chemotherapy Radiotherapy Hormonal therapy Other	64 (70.0) 48 (52.2) 31 (33.7) 18 (19.6) 14 (15.2)		43 (70.5) 33 (54.1) 23 (37.7) 10 (16.4) 10 (16.4)		21 (67.7) 15 (48.4) 8 (25.8) 8 (25.8) 4 (12.9)	
Disease progression at baseline (*n* = 92) Metastasis Relapse	21 (22.8) 11 (12.0)		12 (19.7) 8 (13.1)		9 (29.0) 3 (9.7)	
Education (*n* = 86) University degree Post-school training Still in education Other/no qualification	29 (31.6) 41 (44.6) 3 (3.2) 13 (14.1)		22 (39.3) 22 (39.3) 2 (3.6) 10 (17.9)		7 (23.3) 19 (63.3) 1 (3.3) 3 (10.0)	
Employment (*n* = 86) Full-time Part-time Unemployed	27 (31.4) 12 (14.0) 47 (54.6)		25 (44.6) 11 (19.7) 20 (35.7)		2 (6.7) 1 (3.3) 27 (90.0)	
Marital status (*n* = 87) Married/in a relationship Divorced/seperated Single Widowed	46 (52.9) 11 (12.6) 26 (29.9) 4 (4.6)		30 (52.6) 3 (5.3) 22 (38.6) 2 (3.5)		16 (53.3) 8 (26.7) 4 (13.3) 2 (6.7)	
Lifestyle (*n* = 85) Currently smoking Regular alcohol Vegetarian/vegan nutrition	17 (20.0) 37 (43.5) 5 (5.9)		13 (23.2) 26 (46.4) 2 (3.6)		4 (13.8) 11 (37.9) 3 (10.3)	
Self-evaluation (*n* = 85) Current health (10–100) Current physical performance (10–100)	85 85	65.8 ± 21.1 56.0 ± 21.1	56 56	69.3 ± 19.3 57.7 ± 20.3	29 29	59.0 ± 23.0 52.8 ± 22.5
Physical activity at baseline ^1^ (*n* = 66) Min/week MET min/week Physical activity level (PAL) High PAL Moderate PAL Low PAL WHO recommendations^2^ (%) Fulfilled Not fulfilled	66 66 36 (54.5) 23 (34.8) 7 (10.6) 50 (75.8) 16 (24.2)	984.2 ± 740.0 4022.3 ± 3071.3	49 49 30 (61.2) 16 (32.7) 3 (6.1) 41 (83.7) 8 (16.3)	1057.2 ± 764.8 4404.6 ± 32062.3	17 17 6 (35.3) 7 (41.2) 4 (23.5) 9 (53.9) 8 (47.1)	773.8 ± 618.2 2920.2 ± 2396.5
Wearable activity tracker^3^ (*n* = 61) Steps^4^ per day Active minutes^5^ total Active minutes^5^ light Active minutes^5^ moderate Active minutes^5^ vigorous Active minutes^5^ ≥ moderate			34 34 34 34 34 34	10723.9 ± 4304.4 2258.5 ± 663.2 1836.3 ± 579.1 212.5 ± 123.6 231.2 ± 116.7 443.7 ± 187.0		

^1^Measured by the International Physical Activity Questionnaire – Long form (IPAQ-L).

^2^WHO recommendations met with at least 150 minutes moderate/75 minutes vigorous activity per week.

^3^Measured by Fitbit Charge 2 between baseline (V0) and after six months of intervention (V1); only data included with at least 50% valid weeks.

^4^Steps are reported as the mean per day.

^5^Active minutes are reported as the mean of the sum per week.

SD: standard derivation; TE: telemedicine-based exercise intervention; RG: rehabilitation sports group; MET: Metabolic equivalent of task; BMI: Body mass index; WHR: Waist-hip ratio; PAL: Physical activity level.

### Cardiopulmonary fitness

At baseline, the overall study sample showed low cardiopulmonary fitness with measured values of 1846.6 ml/min with a SD of 604.5 ml/min (absolute VO_2_peak) and 22.9 ml/min/kg with a SD of 7.2 ml/min/kg (relative VO_2_peak). The men of the total study sample (*n* = 52; 53.8 ± 18.4 years old) had a mean absolute VO_2_peak of 2068.3 ml/min with a SD of 628.3 ml/min and a relative VO_2_peak of 23.3 ml/min/kg with a SD of 7.4 ml/min/kg. The women of the total sample of this study (*n* = 37; 48.9 ± 12.8 years old) had measured values of 1535.0 ml/min with a SD of 403.3 ml/min (absolute VO_2_peak) and 22.3 ml/min/kg with a SD of 6.9 ml/min/kg (relative VO_2_peak). Within the study groups, patients of TE had a higher VO_2_peak at baseline (absolute: 2039.8 ± 621.9 ml/min; relative: 25.2 ± 7.3 ml/min/kg) than patients of RG (absolute: 1466.5 ± 331.7 ml/min; relative: 18.3 ± 4.1 ml/min/kg).

**Fig. 2:**
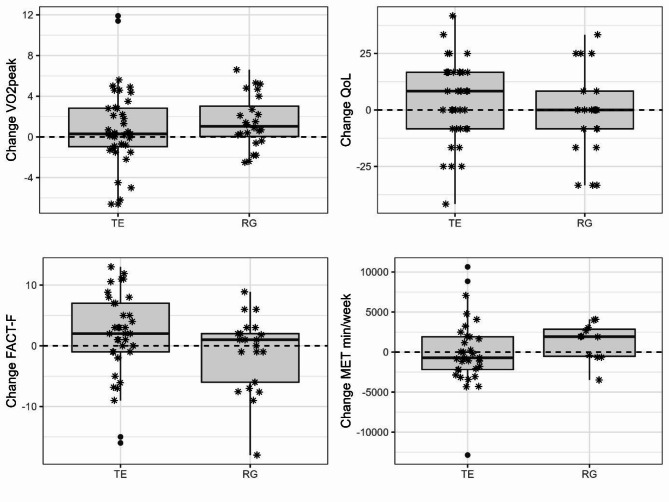
Mean changes of cardiopulmonary fitness, quality of life, fatigue and physical activity by treatment group between baseline (V0) and after six months intervention (V1).

TE: Telemedicine-based exercise intervention; RG: Rehabilitation sports group; ⁕ individual data points; ● outliers.

Figure 2 shows the differences in relative VO_2_peak as V1 – V0 by treatment group. The VO_2_peak of the TE patients increased by 56.3 ± 335.0 ml/min or 1.0 ± 6.4 ml/min/kg between baseline (V0) and after six months intervention (V1). In patients who participated in the rehabilitation sports group, the VO_2_peak increased by 104.0 ± 186.0 ml/min or 2.0 ± 3.9 ml/min/kg. For the non-inferiority-analysis, the adjusted values were used. This results in an adjusted mean difference of 0.55 ml/min/kg [95% CI: − 2.74; 3.84] between the two groups, i.e. TE shows a better improvement than RG. However, the predefined non-inferiority margin is -1.50 ml/min/kg. As the lower end of the CI is below the predefined margin, the non-inferiority of TE compared to RG for relative VO_2_peak cannot be significantly demonstrated (see Table 2).

### Secondary outcomes

The total study population showed an overall QoL of 56.0 ± 22.0 points on a scale of 0 to 100, which was higher in TE (58.8 ± 19.0) than in RG (50.3 ± 26.8) (see Supplementary Appendix III). Patients in the telemedicine-based exercise intervention improved their overall QoL by 4.9 ± 17.6 points, whereas the QoL of patients in RG decreased by 1.6 ± 18.9 points (see Fig. 2). The non-inferiority analysis showed an adjusted mean difference of 10.7 points [95% CI: 0.12; 21.4]. With a predefined margin of 5 points, the telemedicine-based exercise intervention is therefore not inferior to a rehabilitation sports group for improving QoL (see Table 2). In addition, the following functional subscales of QoL improved in patients of TE: Social functioning score improved by 10.7 ± 30.5 points, cognitive functioning score by 9.4 ± 27.5 points, physical functioning score by 2.6 ± 8.7 points and role functioning score by 1.3 ± 30.0 points. The emotional functioning score on the other hand decreased in TE by 2.6 ± 20.7 points on a scale of 0 to 100. Patients participating in the rehabilitation sports group improved their social functioning score by 9.5 ± 24.5 points and their cognitive functioning score by 1.6 ± 13.9 points, but decreased their emotional functioning score by 2.0 ± 18.2 points, their physical functioning score by 0.8 ± 13.9 points and their role functioning score by 0.8 ± 25.5 points (see Supplementary Appendix IV).

At baseline, 36% (*n* = 32) of all participants had fatigue symptoms according to the Cella criteria, with a higher proportion in TE (45%; *n* = 25) than in RG (26%; *n* = 7). According to the symptom scale of the QLQ-C30 as well as the scale of the FACT-F, the severity of the symptoms was comparable in both groups (see Supplementary Appendix III). Patients performing individual telemedicine-based exercise showed a decrease in fatigue symptoms of 4.3 ± 20.3 points on the QLQ-C30 symptom scale. The mean change for patients in the rehabilitation sports group was − 6.4 ± 26.0 points (see Fig. 2).

The level of physical activity differed between the two groups. At baseline, 61% (*n* = 30) of TE participants belonged to the high PAL group and only 6% (*n* = 3) belonged to the low PAL group, while in RG the proportion was 35% (*n* = 6) for high PAL and 24% (*n* = 4) for low PAL During the intervention period, the distribution within the two groups changed. After six months of exercise intervention, 57% (*n* = 20) of TE remained in the high PAL group, while in RG the proportion increased to 67% (*n* = 10). A similar picture emerges when looking at MET minutes per week: Within TE, MET min/week remained unchanged (V0: 4405 ± 3206 MET min/week; V1: 4492 ± 4376 MET min/week). In RG, MET min/week increased by 1307.0 ± 2334.6 MET min/week from 2920 ± 2397 MET min/week to 4178 ± 3415 MET min/week. Analysis of changes in fatigue and physical activity showed no non-inferiority of TE compared to RG (see Table 2).

**Table 2:** Non-inferiority analysis.

	TE	RG	Adjusted Regression Coefficient^1^			Non-inferiority margin
	Change V0 ◊ V1	Change V0 ◊ V1				
	Mean ± SD	Mean ± SD	n	Mean	CI (95%)	
Primary Endpoint^2^ VO_2_peak (ml/min/kg)	1.0 ± 6.4	2.0 ± 3.9	92	0.55	− 2.74; 3.84	− 1.50 ml/min/kg
Secondary Endpoints Quality of Life (Overall Score 0-100) ^3^ Fatigue (FACT-F Score 0–52) ^4^ Physical Activity (MET min/week)	4.9 ± 17.6 1.7 ± 6.9 0.7 ± 4301.9	− 1.6 ± 18.9 − 1.1 ± 6.2 1307.0 ± 2334.6	60 58 43	10.7 3.21 2394	0.12; 21.4 − 0.26; 6.69 − 1032; 5821	− 5 points 3 points − 720 MET min/week

^1^estimates the difference in mean changes of the two groups between baseline (V0) and after 6-months intervention period (V1).

^2^based on adjusted values including multiple imputation.

^3^high score represents high QoL.

^4^high score represents more severe fatigue.

## Discussion

This study compared the effect of a telemedicine-based exercise intervention using wearable activity trackers (AT) on cardiopulmonary fitness with standard care consisting of existing rehabilitation sports groups. While previous studies have used AT only as a tool to improve PA^33^, this study used AT as an integral part of the exercise intervention to improve CS’s physical performance.

The overall cardiopulmonary fitness of this study group was poor. Compared to a healthy sample^47^, the VO_2_peak of the men was just above the 5th percentile. The women are slightly better, but also only between the 10th and 20th percentile. Previous reviews and meta-analyses have shown an association between cardiopulmonary fitness and cancer mortality. Schmid and Leitzmann conclude that increased cardiopulmonary fitness is a strong predictor of reduced risk of cancer mortality^48^. Garcia et al. also highlight the significant protection of physical activity for several chronic disease outcomes in CS^14^. This highlights the importance of regular exercise and physical activity to improve cardiopulmonary fitness in cancer survivors after treatment. In this study, both interventions showed only negligible effects on the cardiopulmonary fitness in cancer survivors. This contradicts the results of previous exercise intervention studies in cancer survivors^49^ and raises the question of reasons. Garber et al. emphasize, that the total amount of exercise, as well as a minimum intensity, is critical for improvements in VO_2_peak and other physiological parameters^50^. The overall goal in both interventions was to meet the WHO recommendations of at least 150 min of moderate or 75 min of vigorous physical activity per week. In the individual telemedicine group, the training plan was individually tailored to the patient’s fitness level at enrollment and previous exercise experience. This approach meant that for individual patients, the initial training plan could start at less than 150 min per week and increase to 150 min per week over the course of the intervention period. For the group intervention, the challenge was to find a rehabilitation sports group that met more than once a week for 45 to 60 min. In summary, the amount of exercise in our interventions did not appear to be sufficient to demonstrate measurable effects on cardiopulmonary fitness. Another reason for the lack of the expected improvement in VO2peak may be the high level of downtime experienced by cancer survivors. Cancer survivors are more susceptible to infections^51^, which interfere with regular exercise. This makes it more difficult to improve cardiopulmonary fitness. Overall, adherence is essential. The analysis of the current sample revealed an adjusted mean difference between the two groups favoring TE over RG, although not statistically significant. The descriptive analysis of the results shows that the mean changes within both groups are accompanied by very high standard deviations. While some patients’ VO_2_peak decreased during the intervention, others seemed to benefit substantially from the exercise intervention. For example, three CS in the telemedicine group improved their cardiopulmonary fitness by 25 to 30%, and in the standard care group, the relative VO_2_peak of two cancer survivors increased by more than 30%. The different responses of individual CS to the interventions support our assumption that an individualized program of endurance and resistance training must be found for each individual cancer survivor that fits the CS’s current physical functioning, prior experience, and interests.

Compared to reference QoL scores^52^, the present sample showed comparable results, although CS in RG showed slightly lower overall QoL scores. While QoL improved in TE patients, a slight deterioration was observed in RG patients. The analysis showed the non-inferiority of TE compared to RG for overall QoL. A telemedicine-based exercise intervention is therefore suitable as an alternative to standard of care in CS. Looking at the functional subscales also reveals some interesting points. The physical functioning subscale showed only small changes in both groups, with a slight decrease in RG. These findings are consistent with the objective VO_2_peak measures. The psychosocial dimensions of QoL, especially social functioning, improved during the intervention period. This scale takes into account the impact of the diagnosis on family and social life. Interestingly, social functioning improved in both groups, although not significantly. This is interesting because TE focuses on individual training that can be done alone, while the RG relies on group training. It is therefore concluded that exercise itself has a positive effect on social functioning, regardless of whether it is done alone or in a group. Overall, assessments of QoL need to take into account the context of the global COVID-19 pandemic. A survey by Bargon et al. of more than 1000 breast cancer patients shortly after the start of the pandemic showed a significant deterioration in emotional functioning^53^. The impact of pandemic-related restrictions and burdens, particularly on the psychosocial aspects of quality of life, cannot be clearly defined in this study. Therefore, statements regarding the impact of the exercise interventions on QoL are limited.

The beneficial effects of physical activity and exercise on fatigue are well established^1,54^. In this study, both interventions led to a small improvement in fatigue symptoms. The time since the initial diagnosis may have played a role here, as the symptoms improve in the months following completion of therapy^54^. Time since diagnosis showed a wide range for the individual CS in this study. Some patients were only six weeks after completion of acute therapy, while others have been diagnosed for several years. However, based on the non-inferiority analysis, no definitive conclusions can be drawn about the differences between the two interventions.

Previous studies have shown that ATs are effective tools for increasing physical activity^33,55^. Although there were notable differences in the changes in physical activity levels between the two groups in this study, these findings were not replicated. In contrast, the physical activity level of CS in the telemedicine group remained almost the same. At the same time, there was a substantial increase in the standard care group. The weekly MET minutes in this group increased by 40%, and the proportion of patients with high activity levels increased from one third at baseline to two thirds after six months of intervention. When interpreting the physical activity, it is important to consider baseline activity levels^50^. The fact that the participants in the telemedicine group differed from those in the control group in terms of age, physical activity level, and cardiopulmonary fitness at the start of the study seems to have played an important role. This suggests that CS with low activity levels may particularly benefit from a supervised exercise intervention. Despite the pandemic that led to the temporary suspension of the group interventions, the link with sports medicine, counseling and referral to an exercise group alone appears to influence awareness of the importance of physical activity. The reasons for the consistent activity levels in the telemedicine group are difficult to determine at this stage. It is likely that this consistency is related to the higher activity levels at baseline. At baseline, already 84% of patients in TE met the WHO criteria. They also took more than eleven thousand steps per day during the first six months of the intervention. These levels are remarkably high compared to other studies of CS^56^.

The free choice of intervention provides insight into cancer survivors’ preferences for different exercise options. The fact that two thirds of all participants chose the telemedicine-based exercise option instead of participating in an existing rehabilitation sports group, shows the attractiveness of technology-assisted exercise interventions. When comparing the two groups, it is noticeable that the TE participants were younger than the RG participants. About 40% of the TE patients were under 40 years of age. In RG, however, only one out of thirty-one patients (3%) was younger than 40 years, and almost two-thirds of them were already retired. This is also reflected in the low rate of employment in RG. Nine out of ten patients in RG were not employed; in TE the proportion was only one third, and two thirds were employed part-time or full-time. In conclusion, a telemedicine-based training option seems to be attractive especially for younger, employed patients. The YOUEX study by Voland et al. examined the exercise preferences of young adults with cancer up to the age of 39, including digital exercise interventions. They emphasize the unique life situation of this young cohort, such as their involvement in work and family life, and point out that the most mentioned reason for choosing the individualized online exercise program was time flexibility^57^.

A number of studies have shown that supervised exercise is more effective than home-based exercise^17,58^. However, supervised on-site exercise has the disadvantage of limited availability, particularly in rural areas, and often offers less time flexibility^59^. Developing an individualized exercise plan that can be completed independently at home could be an alternative to supervised programs. Digital media, such as wearable activity trackers, have the advantage that simply wearing a device has a positive effect on physical activity^33,35,36^. The idea of combining an individualized, location- and time-flexible training plan with the use of AT seems both logical and promising in this context. The aim of this study was therefore to use AT to support, monitor, and adjust independent exercise. Regular contact by telephone and e-mail was intended to counteract the inferiority of the home-based intervention.

The implementation of telemedicine-based exercise therapy and close monitoring of exercise plan adherence required patients to wear the AT regularly. The overall wearing behavior of the Fitbit Charge 2 in this study was only 80% of the time. As a result, many of the patients’ activities and exercise sessions were either not recorded or not fully captured by the trackers. These missing data made it difficult for the instructors to monitor the patients. The compliance rate of 80% is lower than that reported in previous studies. For example, Hardcastle et al.^55^ reported a high Fitbit engagement of 94.6% over a 12-week intervention. However, it should be noted that the criteria for a valid day in that study, defined by a minimum step count of 1000 steps per day, were much broader than in this study, where a valid day was defined as at least 10 h of wearing time per day and a valid week was defined as at least 4 valid days. Another challenge in the monitoring was that many CS were difficult to reach by telephone. Despite several attempts back and forth, phone calls were often not made. On average, each patient had contact with the instructor every 4 weeks, although there was considerable variation between patients as well. The highest contact density was 12 times in six months, while other patients had only three telephone contacts during the entire intervention period. It is postulated that the variability in response to supervision may be a potential reason for the variable and inconclusive changes in cardiopulmonary fitness.

However, this is particularly interesting in the context of the COVID-19 pandemic. One of the major advantages of TE over RG is that it was not limited by the COVID-19 restrictions. In theory, close monitoring could have continued if patients had accepted it. The fact that many patients did not accept the offer shows the strong influence of the pandemic itself on individuals. It is therefore interesting to look at the number of dropouts and the reasons for dropping out. The overall dropout rate of 45% in this study is higher than in others^60^. An obvious reason for the high dropout rate in this study could be the COVID-19 pandemic, although on closer inspection only a quarter of the dropouts were related to the pandemic. These pandemic-related dropouts also include cases in which V1 or V2 was not possible due to pandemic-related restrictions regarding the implementation of V1, due to caring responsibilities for relatives or due to the closure of accommodation facilities during the pandemic, so that patients who had to travel long distances could not participate in the examinations. Interestingly, the dropout rate in TE (46%) is even higher than in RG (36%). In addition to reasons such as relapse or personal reasons in both groups, the reason ‘no response’ appears only in the telemedicine group. A total of nine patients from the TE group could no longer be reached. In the RG, all patients could be contacted by the end of the study or gave a reason for dropping out. It is assumed that telemedicine, although as close as possible, was somewhat less personal than a face-to-face training in a group that meets weekly at a fixed place and time. Therefore, the hurdle for dropping out may be lower in TE than in RG. In conclusion, there are large differences between individuals in whether and how they have accepted the telemedicine-based exercise intervention. To gain a deeper insight into this aspect, future studies should also examine the aspect of patient satisfaction. At this point, it can be concluded that it is important to identify the most appropriate exercise intervention for each individual cancer patient in order to achieve an effective and lasting effect. As previously stated, adherence and the appropriate volume of exercise are critical. The objective is to identify a program that the individual CS will enjoy and that will maintain their motivation. In order to achieve this, criteria should be developed in the future with the aim of defining these issues in an initial consultation and selecting the appropriate program. Moreover, further studies with TE should be conducted to investigate the key advantages of TE, such as independence from place and time, as well as the possibility of an immediate start in the increasingly digitalized healthcare system. At the same time, it should be considered whether the high expenditure of time and personnel required for telemedicine^28^ can be reflected in the current healthcare system. Future studies of telemedicine-based interventions should include a cost-benefit-analysis to gain knowledge about the financial aspects in addition to the clinical outcomes. In addition, other remote approaches such as app-based exercise programs or hybrid models may be considered in future studies.

The main strength of this study is its real-world relevance. Due to the lack of comprehensive coverage of rehabilitation sports groups in rural areas, randomization was not practical in this study. Therefore, participants were able to choose their preferred type of exercise. This allowed the preferences and previous experience of the participants to be considered. On the other hand, the lack of randomization results in a high heterogeneity of the two study groups, both in terms of the sample size and their characteristics. This made the groups less comparable and required adjustment for confounders. The small overall sample size is also a limitation, which is exacerbated by the high dropout rate. The active control group is based on existing rehabilitation sports groups, which avoids the need to create new structures and operates in a real-world setting. At the same time, the study coordinators had no influence on the specific content of the group and did not receive detailed information about it, which led to limited standardization of the training. In addition, due to COVID-19 restrictions, many rehabilitation sport groups could not take place for several weeks. TE patients followed a personalized exercise plan that varied in volume, intensity, and the type of exercise. This allowed the training to be tailored to the specific needs and condition of each CS. On the other hand, there was also limited standardization of training in TE, which makes comparisons difficult. Future studies should use more standardized protocols to improve comparability and reproducibility. On top of that, there is a lack of data on adherence. This makes it difficult to draw clear conclusions about the effect of the intervention. The interpretation of activity levels is based on self-reported questionnaire data only. Data from the IPAQ-L questionnaire resulted in many missing values in this study due to the complexity of the questionnaire, which reduces the robustness of the findings. Another limitation is the possibility of (self-) selection bias, which is a common problem in studies of exercise interventions^61^. In addition, this study recruited CS exclusively from a single health insurance company, which introduces a potential recruitment bias.

In conclusion, the implementation of a telemedicine-based exercise intervention suggests that individual patients respond well to this type of exercise program and benefit from the intervention. In particular, the quality of life of cancer survivors is positively influenced by the intervention. Therefore, a telemedicine-based exercise intervention could be a viable alternative to standard care, especially for younger patients. The considerable heterogeneity of the patients in this study and their different responses to the intervention highlight the importance of tailoring the exercise offer to the specific needs and preferences of each individual cancer survivor. Future research should focus on developing criteria that predict whether individual patients will accept and adhere to telemedicine-based exercise therapy. Only then can cancer survivors benefit from the exercise program, including improvements in cardiopulmonary fitness. This will require further intervention studies of telemedicine-based exercise therapy and analysis of adherence data from cancer survivors to exercise interventions.

## Electronic supplementary material

Below is the link to the electronic supplementary material.

Supplementary Material 1

Supplementary Material 2

Supplementary Material 3

Supplementary Material 4

## Data Availability

The datasets generated and analysed during the current study are available from the corresponding author on reasonable request.
