# Supplementary material for: Telemedicine-based exercise intervention in cancer survivors: a non-randomized controlled trial
Source: Sci Rep. 2024 Dec 23;14:30615. doi: 10.1038/s41598-024-83846-x (PMC11666603; doi:10.1038/s41598-024-83846-x)

Directed Acyclic Graph (DAG)

Outcome: VO_2max

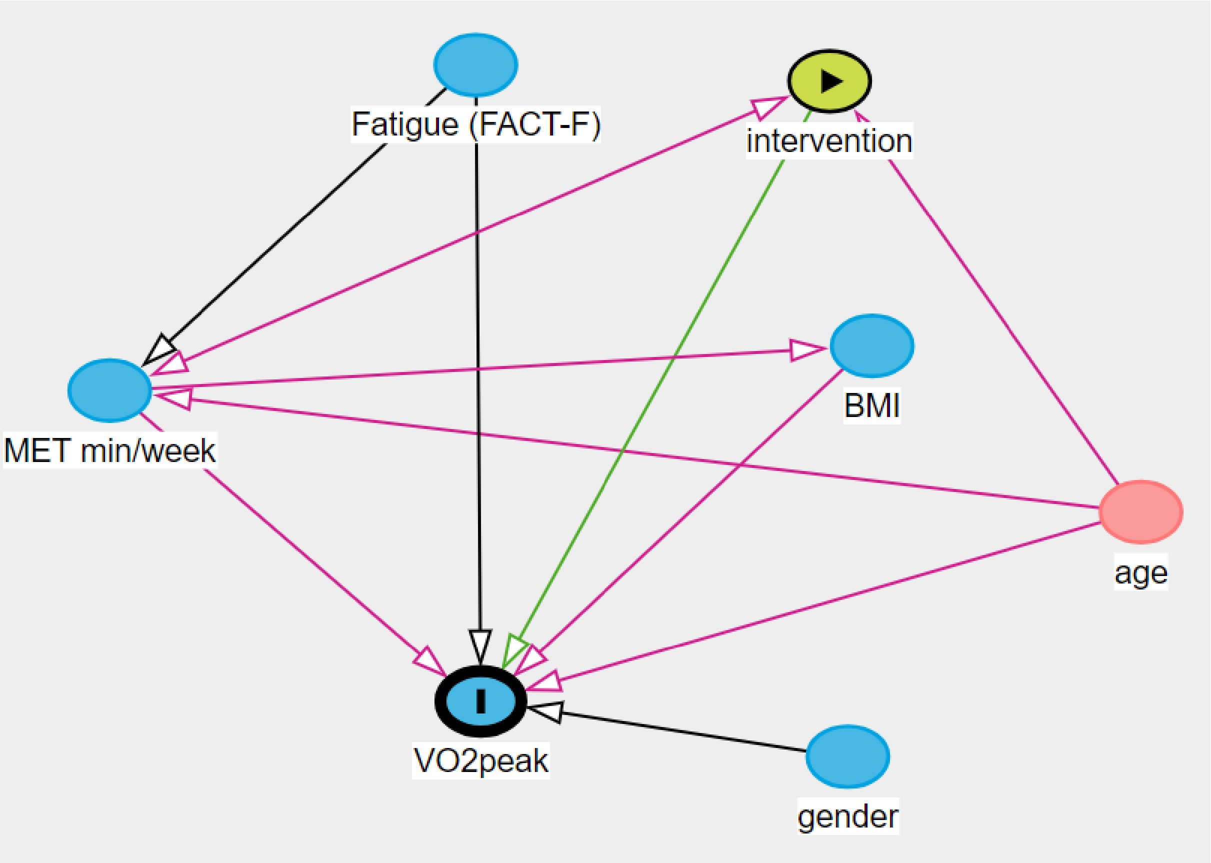

Outcome: Quality of Life

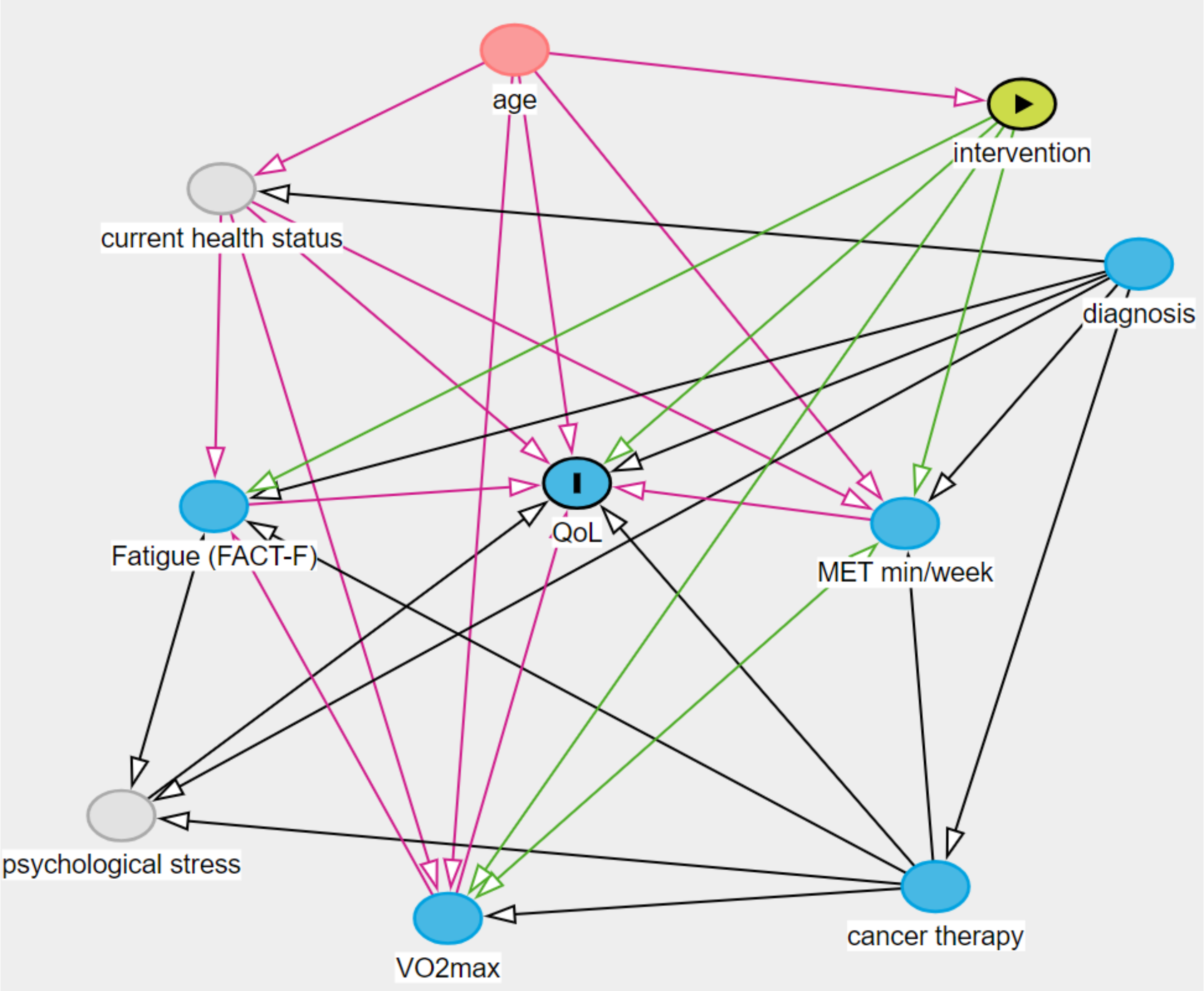

Outcome: Fatigue (FACT-F score)

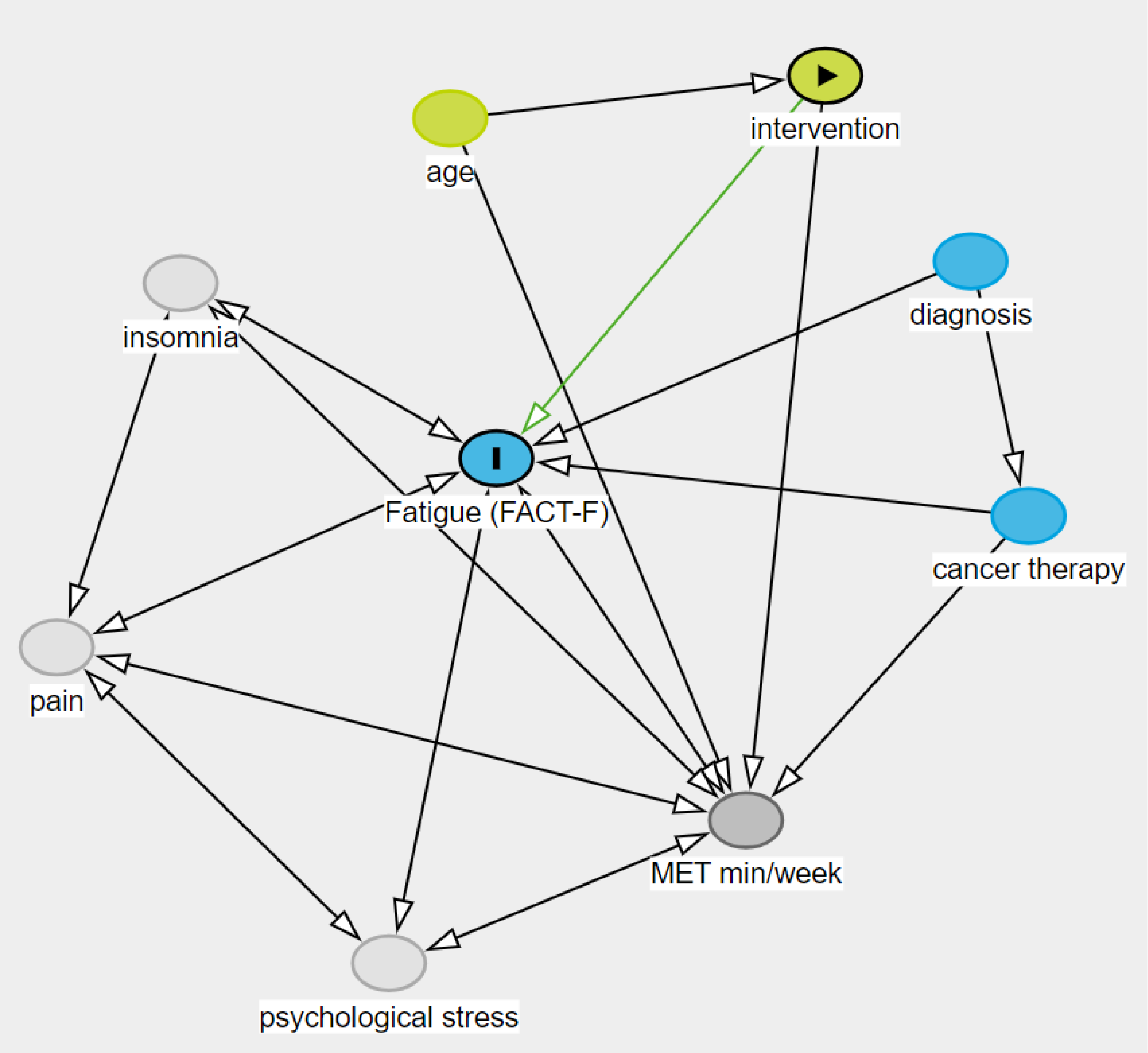

Outcome: Physical Activity

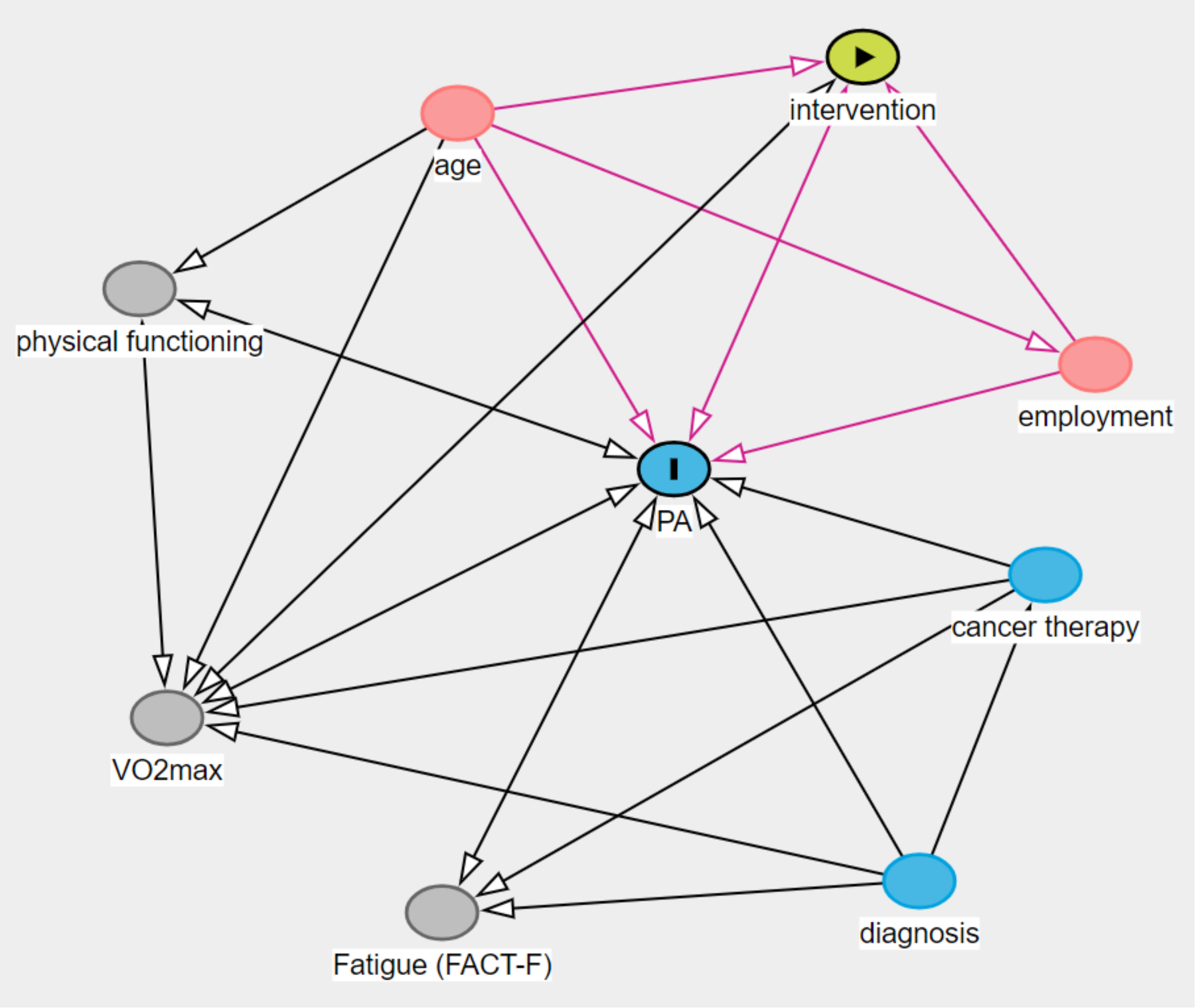

Supplement: Supplementary file 1 — Supplementary Material 1 [file 41598_2024_83846_MOESM1_ESM.pdf]
