# Supplementary material for: Telemedicine-based exercise intervention in cancer survivors: a non-randomized controlled trial
Source: Sci Rep. 2024 Dec 23;14:30615. doi: 10.1038/s41598-024-83846-x (PMC11666603; doi:10.1038/s41598-024-83846-x)
Supplement: Supplementary file 2 — Supplementary Material 2 [file 41598_2024_83846_MOESM2_ESM.pdf]

List of variables used for multiple imputation:

- Intervention („gruppe")
- Age at baseline („alter_v0")
- Gender („geschlecht")
- Place of residence („wohnort")
- BMI at baseline („bmi_v0")
- Body fat mass at baseline („bfm_proz_v0")
- Time since diagnosis (months) at baseline („an_diag1_mon_seit_ed")
- Employment at baseline („an_beruf")
- Marital status at baseline („an_fam_stand")
- Smoking behavior at baseline („an_rau_akt")
- Alcohol consumption at baseline („an_alk_akt")
- Nutrition status at baseline („an_ern_veget")
- Current health (self-assessment) at baseline („an_akt_gesundh")
- Current fitness (self-assessment) at baseline („an_akt_leistungsf")
- Cardiopulmonary fitness: Relative VO_2peak at baseline ("vo2maxkg_v0_plaus")
- Cardiopulmonary fitness: Relative VO_2peak at V1 ("vo2maxkg_v1_plaus")
- Cardiopulmonary fitness: Change of relative VO_2peak between V0 and V1 ("dif_vo2max_kg")
- Cardiopulmonary fitness: Peak power in watts at baseline („p_max_v0")
- Cardiopulmonary fitness: Maximum ventilation at baseline ("ve_at_max_v0")
- QoL: Global health status at baseline („qo_l_v0")
- QoL: Physical functioning at baseline ("qlq_physical_v0")
- QoL: Emotional functioning at baseline („qlq_emotional_v0")
- QoL: Cognitive functioning at baseline ("qlq_cognitive_v0")
- QoL: Emotional functioning at baseline ("qlq_social_v0")
- Fatigue: FACT-F score at baseline („fact_f_v0")
- Fatigue: Cella criteria at baseline („cella_krit_v0")
- Fatigue: QLQ-C30 fatigue symptom score at baseline ("qlq_fatigue_v0")
- PA at baseline (IPAQ MET min/week) ("ipaq_tot_intensity_met_v0")
- PA between 20 and 29 years ("an_pa_20_29")
- PA at baseline during work ("an_pa_work")
