# Supplementary material for: Telemedicine-based exercise intervention in cancer survivors: a non-randomized controlled trial
Source: Sci Rep. 2024 Dec 23;14:30615. doi: 10.1038/s41598-024-83846-x (PMC11666603; doi:10.1038/s41598-024-83846-x)
Supplement: Supplementary file 3 — Supplementary Material 3 [file 41598_2024_83846_MOESM3_ESM.pdf]

Supplementary Appendix III: Descriptive Outcome Measures

	Total						TE						RG					
	V0 (n = 92)		V1 (n = 68)		V2 (n = 53)		V0 (n = 61)		V1 (n = 42)		V2 (n = 33)		V0 (n = 31)		V1 (n = 26)		V2 (n = 20)	
	n	Mean ± SD<br>n (%)	n	Mean ± SD<br>n (%)	n	Mean ± SD<br>n (%)	n	Mean ± SD<br>n (%)	n	Mean ± SD<br>n (%)	n	Mean ± SD<br>n (%)	n	Mean ± SD<br>n (%)	n	Mean ± SD<br>n (%)	n	Mean ± SD<br>n (%)
Cardiopulmonary fitness																		
VO_2peak (ml/min/kg)	89	22.9 ± 7.2	65	24.3 ± 7.6	52	24.0 ± 7.7	59	25.2 ± 7.3	41	26.9 ± 7.8	32	26.1 ± 8.0	30	18.3 ± 4.1	24	19.9 ± 4.7	20	20.6 ± 6.0
VO_2peak (ml/min)	89	1846.6 ± 604.5	65	1888.8 ± 614.4	52	1850.4 ± 637.5	59	2039.8 ± 621.9	41	2092.2 ± 644.1	32	2046.1 ± 658.6	30	1466.5 ± 331.7	24	1541.5 ± 359.5	20	1537.2 ± 464.3
Pmax (W)	91	127.2 ± 48.8	66	132.1 ± 54.0	52	130.4 ± 54.9	60	141.9 ± 50.6	41	151.9 ± 54.4	32	149.5 ± 54.6	31	98.8 ± 29.3	25	99.7 ± 34.7	20	99.9 ± 36.7
Pmax (W/kg)	91	1.58 ± 0.62	66	1.69 ± 0.69	52	1,70 ± 0,71	60	1.76 ± 0.65	41	1.96 ± 0,69	32	1.93 ± 0.74	31	1.22 ± 0.37	25	1.25 ± 0.39	20	1.33 ± 0.47
Quality of Life																		
Overall QoL	83	56.0 ± 22.0	62	60.9 ± 25.1	47	59.2 ± 22.9	56	58.8 ± 19.0	40	65.4 ± 24.8	30	59.7 ± 21.8	27	50.3 ± 26.8	22	52.7 ± 24.0	17	58.3 ± 25.3
Physical functioning	84	80.4 ± 17.3	63	83.7 ± 17.3	49	79.5 ± 19.1	56	83.5 ± 15.0	40	89.5 ± 12.1	31	84.3 ± 17.4	28	74.2 ± 20.1	23	73.6 ± 20.5	18	71.1 ± 19.4
Role functioning	83	78.1 ± 29.2	63	79.1 ± 26.4	48	79.5 ± 29.0	56	80.1 ± 26.7	40	83.3 ± 24.7	31	82.3 ± 27.9	27	74.1 ± 34.1	23	71.7 ± 28.2	17	74.5 ± 31.2
Emotional functioning	83	63.5 ± 27.5	62	64.4 ± 26.9	48	67.5 ± 26.1	56	62.4 ± 27.7	40	64.6 ± 26.3	31	69.6 ± 23.7	27	66.1 ± 27.4	22	64.0 ± 28.6	17	63.7 ± 30.3
Social functioning	83	67.3 ± 32.6	62	79.6 ± 28.5	48	72.2 ± 33.0	56	66.1 ± 31.1	40	80.4 ± 27.5	31	75.8 ± 32.2	27	69.8 ± 35.8	22	78.0 ± 31.0	17	65.7 ± 34.6
Cognitive functioning	83	69.3 ± 28.6	62	77.2 ± 26.5	48	76.7 ± 25.0	56	68.8 ± 27.0	40	79.6 ± 23.1	31	76.9 ± 26.1	27	70.4 ± 32.1	22	72.7 ± 31.9	17	76.5 ± 23.6
Fatigue																		
FACT-F (Score 0-52)	83	34.4 ± 11.6	60	36.7 ± 11.7	48	35.4 ± 12.6	56	34.3 ± 11.1	40	38.4 ± 10.7	31	36.1 ± 12.9	27	35.0 ± 13.0	22	33.7 ± 12.9	17	34.0 ± 12.2
QLQ Fatigue (Score 0-100)	83	43.5 ± 28.9	62	37.8 ± 29.5	48	38.2 ± 30.2	56	42.7 ± 28.0	40	36.9 ± 30.3	31	38.7 ± 31.8	27	45.3 ± 31.1	22	39.4 ± 28.4	17	37.3 ± 28.0
Cella-Criteria fulfilled	83	32 (35.5)	60	15 (25.0)	48	14 (29.2)	56	25 (44.6)	38	8 (21.1)	31	9 (29.0)	27	7 (25.9)	22	7 (31.8)	17	5 (29.4)
Physical Activity																		
Min/week	66	984 ± 740	50	1006 ± 850	38	1031 ± 833	49	1057 ± 765	35	993 ± 884	28	1126 ± 833	17	774 ± 637	15	1037 ± 792	10	726 ± 798
MET min/week	66	4022 ± 3071	50	4398 ± 4079	38	4324 ± 3721	49	4405 ± 3206	35	4492 ± 4376	28	4867 ± 3918	17	2920 ± 2397	15	4178 ± 3415	10	2804 ± 2716
Physical activity level (PAL)	66		50		38		49		35		28		17		15		10	
High PAL		36 (54.5)		30 (60.0)		20 (52.6)		30 (61.2)		20 (57)		16 (57.1)		6 (35.3)		10 (66.7)		4 (40.0)
Moderate PAL		23 (34.9)		14 (28.0)		15 (39.5)		16 (32.7)		11 (31)		10 (35.7)		7 (41.2)		3 (20.0)		5 (50.0)
Low PAL		7 (10.6)		6 (12.0)		3 (7.9)		3 (6.1)		4 (11)		2 (7.2)		4 (23.5)		2 (13.3)		1 (10.0)
