# Supplementary material for: Telemedicine-based exercise intervention in cancer survivors: a non-randomized controlled trial
Source: Sci Rep. 2024 Dec 23;14:30615. doi: 10.1038/s41598-024-83846-x (PMC11666603; doi:10.1038/s41598-024-83846-x)
Supplement: Supplementary file 4 — Supplementary Material 4 [file 41598_2024_83846_MOESM4_ESM.pdf]

Supplementary Appendix IV: Descriptive Statistics of Outcome Measures by Treatment Group at Baseline (V0) and After Six Months Intervention (V1)

	TE					RG				
	V0		V1		Mean Change[1]	V0		V1		Mean Change[1]
	n	Mean ± SD	n	Mean ± SD	Mean ± SD	n	Mean ± SD	n	Mean ± SD	Mean ± SD
Cardiopulmonary fitness										
VO_2peak (ml/min/kg)	59	25.2 ± 7.3	41	26.9 ± 7.8	1.0 ± 6.4	30	18.3 ± 4.1	24	19.9 ± 4.7	2.0 ± 3.9
VO_2peak (ml/min)	59	2039.8 ± 621.9	41	2092.2 ± 644.1	56.3 ± 335.0	30	1466.5 ± 331.7	24	1541.5 ± 359.5	104.0 ± 186.0
Pmax (W)	60	141.9 ± 50.6	41	151.9 ± 54.4	5.7 ± 15.4	31	98.8 ± 29.3	25	99.7 ± 34.7	3.0 ± 11.8
Pmax (W/kg)	60	1.76 ± 0.65	41	1.96 ± 0,69	0.11 ± 0.22	31	1.22 ± 0.37	25	1.25 ± 0.39	0.03 ± 0.15
Quality of Life										
Overall QoL	56	58.8 ± 19.0	40	65.4 ± 24.8	4.9 ± 17.6	27	50.3 ± 26.8	22	52.7 ± 24.0	- 1.6 ± 18.9
Physical functioning	56	83.5 ± 15.0	40	89.5 ± 12.1	2.6 ± 8.7	28	74.2 ± 20.1	23	73.6 ± 20.5	- 0.8 ± 13.9
Role functioning	56	80.1 ± 26.7	40	83.3 ± 24.7	1.3 ± 30.0	27	74.1 ± 34.1	23	71.7 ± 28.2	- 0.8 ± 25.5
Emotional functioning	56	62.4 ± 27.7	40	64.6 ± 26.3	- 2.6 ± 20.7	27	66.1 ± 27.4	22	64.0 ± 28.6	- 2.0 ± 18.2
Social functioning	56	66.1 ± 31.1	40	80.4 ± 27.5	10.7 ± 30.5	27	69.8 ± 35.8	22	78.0 ± 31.0	9.5 ± 24.5
Cognitive functioning	56	68.8 ± 27.0	40	79.6 ± 23.1	9.4 ± 27.5	27	70.4 ± 32.1	22	72.7 ± 31.9	1.6 ± 13.9
Fatigue										
FACT-F (Score 0-52)	56	34.3 ± 11.1	40	38.4 ± 10.7	1.7 ± 6.9	27	35.0 ± 13.0	22	33.7 ± 12.9	- 1.1 ± 6.2
QLQ Fatigue (Score 0-100)	56	42.7 ± 28.0	40	36.9 ± 30.3	- 4.3 ± 20.3	27	45.3 ± 31.1	22	39.4 ± 28.4	- 6.4 ± 26.0
Physical Activity										
Minutes per week	49	1057 ± 765	35	993 ± 884	- 89.4 ± 901.6	17	774 ± 637	15	1037 ± 792	284.1 ± 591.7
MET min/week	49	4405 ± 3206	35	4492 ± 4376	0.7 ± 4301.9	17	2920 ± 2397	15	4178 ± 3415	1307.0 ± 2334.6

[1]*Mean Change (V0 – V1)*
